# Supplementary material for: MetaRibo-Seq measures translation in microbiomes
Source: Nat Commun. 2020 Jun 29;11:3268. doi: 10.1038/s41467-020-17081-z (PMC7324362; doi:10.1038/s41467-020-17081-z)
Supplement: Supplementary file 10 — Supplementary Data 7 [file 41467_2020_17081_MOESM10_ESM.zip › File2/Confidence_High_Taxonomy/276046_out.krona.html]

Javascript must be enabled to view this page.

members
magnitude
magnitudeUnassigned
count
unassigned
taxon
rank

276046\_out

3

3
2
superkingdom

976
phylum
3

3
200643
class

order
171549
3

815
family
3

3
816
genus

species
671267
2

SRS024388\_contig\_number\_14691SRS1041132\_contig\_number\_8139


SRS011302\_contig\_number\_10357
821
species
1
